# Roles of microRNAs and histone modifications in enhancing stress tolerance in soybean and their applications in molecular breeding

**DOI:** 10.1270/jsbbs.24039

**Published:** 2025-02-21

**Authors:** Kejing Fan, Long-Yiu Chan, Sze-Wing Chan, Wai-Shing Yung, Liping Wang, Hui Zhang, Hon-Ming Lam

**Affiliations:** 1 School of Life Sciences and Centre for Soybean Research of the State Key Laboratory of Agrobiotechnology, The Chinese University of Hong Kong, Shatin, Hong Kong SAR, P. R. China

**Keywords:** soybean, microRNA (miRNA), histone modification, genomics, molecular breeding, biotic stress, abiotic stress

## Abstract

Soybean is an ancient crop domesticated from wild soybean (*Glycine soja* Sied. & Zucc) in East Asia 6,000–9,000 years ago and has been widely grown as human food and livestock feed in China, Korea, Japan, and the rest of the world since. Global climate change has led to a series of challenges in soybean cultivation and breeding. With the development of high-throughput genomic sequencing technologies, genomic information on soybeans is now more readily available and can be useful for molecular breeding. However, epigenetic regulations on crop development are still largely unexplored. In this review, we summarized the recent discoveries in the regulatory mechanisms underlying soybean adaptations to biotic and abiotic stresses, particularly with respect to histone modifications and microRNAs (miRNAs). Finally, we discussed the potential applications of this knowledge on histone modifications and miRNAs in soybean molecular breeding to improve crop performance in the changing environment.

## Introduction

With an increasing world population, more frequent epidemics, and escalating regional conflicts, more efficient and sustained crop production is required worldwide. However, climate change further aggravates the challenges to the global food system. Maintaining genetic diversity and crop breeding are key strategies for combating the effects of climate change. Soybean is one of the most important crops globally, and provides oil and protein for both humans and livestock. World soybean production in 2022 was approximately 348.9 million tons, grown on more than 133.8 million hectares (FAOSTAT, www.fao.org). However, soybean growth is easily impacted by environmental stresses. For example, global soybean production is estimated to decrease by 3.1% for each further one degree Celsius increase in global temperature (Zhao *et al.* 2017). To sustain soybean production under extreme environmental stresses, effective molecular breeding technologies are in urgent need.

MicroRNAs (miRNAs) and histone modifications are two underrated genetic resources in the current soybean molecular breeding processes. MiRNA, a small non-coding RNA, negatively regulates its target gene expression at the post-transcriptional level (Fan *et al.* 2022). MiRNAs are usually transcribed by RNA polymerase II into primary-miRNA (pri-miRNA), which are then cleaved by dicer-like proteins to form mature miRNAs. MiRNAs combine with Argonaute proteins (AGOs) to form a multiprotein RNA-induced silencing complex (RISC), whose function is to silence its target gene via miRNA guidance at the post-transcriptional and translational levels (reviewed by Song *et al.* 2019). MiRNAs are an evolutionarily conserved class of small RNAs with conserved target gene families in plants. Conserved miRNAs are usually ancient miRNAs which control the expressions of genes that are fundamental to the process of life across most plant species. There are also many unconserved miRNAs in each plant species, making it possible for miRNA regulation to extend into virtually every aspect of plant biology (Millar 2020). MiRNAs have been shown to control soybean growth, development, stress responses, and symbiosis with rhizobia (Fan *et al.* 2021, 2023, Kumar 2014). Extensive small-RNA sequencing analyses of different soybean tissues under various stresses were conducted in the last decade to identify miRNAs that are responsive to environmental stimuli, providing valuable genetic resources for soybean breeding research.

In eukaryotes, DNA wraps around octamers of histone proteins (two each of H2A, H2B, H3, and H4) to form a series of nucleosomes, the basic units of chromatin. (Dong and Weng 2013). Chromatin contains the template of transcription. Actively transcribed regions usually have relaxed structures so that transcription factors and RNA polymerases can access and bind to the stretch of DNA containing the genetic information. The chromatin structure can be altered by histone modifications, which in turn can modify the rate of gene transcription (Dong and Weng 2013). Histone proteins are subjected to different post-translational modifications, such as acetylation and methylation. These modifications are functionally important in transcriptional regulation by altering the chromatin structure to either facilitate or inhibit access by the transcriptional machinery or as marks to be recognized by specific readers involved in transcription (Bannister and Kouzarides 2011).

When under stress, plants carry out extensive transcriptional reprogramming to maximize their survival and well-being. Therefore, the integration of miRNAs and histone modifications into soybean breeding strategies could be an effective approach to maintaining sustainable soybean production.

## Effects of microRNAs (miRNAs) on soybean stress tolerance

MiRNAs are key regulators of soybean growth and are responsive to many biotic and abiotic stresses (Table 1). In this section, recent findings on miRNA regulatory roles in soybean stress tolerance are discussed.

### Biotic stress

Biotic stress is an adverse condition in which plants cannot maintain their normal growth when encountering deleterious organisms such as fungi, viruses, bacteria, as well as nematodes and phytoplasma. It is a critical factor limiting soybean growth and yield (Šečić *et al.* 2021).

Plant miRNAs were reported to play significant roles in plant defense against insect herbivory by targeting either the defense-responsive genes in the plant or the genes of herbivorous insects (Bordoloi and Agarwala 2021). Bean pyralid (*Lamprosema indicata* [Fabricius], Family Pyralidae, Order Lepidoptera) is a major leaf-feeding pest of soybean (Zeng *et al.* 2019). To screen for the functional soybean miRNAs related to the resistance against bean pyralid larvae, small-RNA sequencing was performed using a resistant soybean line ‘Gantai-2-2’ and a susceptible line ‘Wan82-178’ (Zeng *et al.* 2019). When comparing ‘Wan82-178’ to ‘Gantai-2-2’, 77 differentially expressed (DE) miRNAs were identified at 0-h feeding, and 70 DE miRNAs were found at 48-h feeding (Zeng *et al.* 2019). Among these DE miRNAs, miR156, miR166, miR319, miR394, and miR396, together with their negatively regulated differentially expressed target genes, may be involved in regulating soybean resistance to bean pyralid larvae (Zeng *et al.* 2019).

Infection by soybean mosaic virus (SMV) strain ‘G7’ led to the accumulation of miR168 and the *AGO1* (*ARGONAUTE1*) mRNA (Chen *et al.* 2015). An elevated miR168 level repressed the translation of the *AGO1* protein, disrupting *AGO1* homeostasis (Chen *et al.* 2015). Silencing *SGS3* (*Suppressor of Gene Silencing 3*), an essential component in RNA silencing, could reduce the level of AGO1-derived siRNAs, partially reverse the repression of AGO1 protein expression, and alleviate SMV G7 infection symptoms (Chen *et al.* 2015). This evidence hinted that both miRNA and siRNA pathways are associated with SMV G7 pathogenesis in soybean (Chen *et al.* 2015).

Upon infection by the fungus *Phytophthora sojae* (*P. sojae*), the second most destructive pathogen of soybean, miR166, miR393, miR1507, miR2109, and miR3522 were induced, but miR168, miR319 and miR482 levels were decreased (Wong *et al.* 2014). The induction of miR393 could be triggered by heated-inactivated *P. sojae* and positively regulated soybean defense against the pathogen by modulating the isoflavonoid biosynthetic pathway that governs the production of antimicrobial metabolites (Wong *et al.* 2014). In contrast to miR393, miR1510 was downregulated during *P. sojae* infection and was shown to target a plant disease resistance gene containing both the toll interleukin-like receptor (TIP) and nucleotide-binding site-leucine-rich repeat (NB-LRR) domains, while the overexpression of miR1510 significantly reduced the soybean resistance to *P. sojae* (Cui *et al.* 2017).

Soybean cyst nematode (SCN, *Heterodera glycines*), the world’s most significant pathogen of soybean, could penetrate soybean roots and migrate into the vascular bundle where it forms a feeding site syncytium (Noon *et al.* 2019). Many miRNAs have been reported to control the soybean resistance to *H. glycines*, such as miR396 and miR159 (Lei *et al.* 2021, Noon *et al.* 2019). The miR396 expression showed dynamic changes during syncytium development, acting as a master regulator of SCN infection in Arabidopsis (Hewezi *et al.* 2012). During syncytium formation, miR396 was downregulated, resulting in the induction of its target genes, *GRF*s (*GROWTH-REGULATING FACTOR*). The overexpression of miR396 and knocking down of *GRF*s inhibited nematode development and resulted in reduced syncytium size (Hewezi *et al.* 2012). After infection by SCN, 7,225 differentially expressed (DE) genes were found in Arabidopsis syncytia (Szakasits *et al.* 2009). The overexpression of *GRF*s could alter the expression of 44% of the 7,225 DE genes. Therefore, the miR396-*GRF* module plays a regulatory role in plant defense against SCN infection (Hewezi *et al.* 2012). However, during the syncytium maintenance phase, miR396 was upregulated, leading to the downregulation of *GRF*s (Noon *et al.* 2019). Interference with the accumulation of miR396 through overexpression or knockdown mutation did not affect the formation of syncytia but it inhibited their maturation, hinting that the miR396-*GRF* module is essential for effective *H. glycines* infections (Noon *et al.* 2019).

*H. glycines* infection also induced different methylation levels in the miRNA promoters in susceptible versus resistant isogenic soybean lines, especially those of miR5032, miR5043, miR1520b, and miR5770a/b (Rambani *et al.* 2020). The overexpression of these four miRNAs in susceptible isogenic lines enhanced the soybean resistance to SCN to varying degrees (Rambani *et al.* 2020). This crosstalk between miRNA and DNA methylation provided new insights into the role of epigenetic mechanisms in controlling soybean stress tolerance.

### Abiotic stress

Abiotic stresses on plants are caused by the negative effects of non-living elements in the environment (de Lima *et al.* 2012, Yung *et al.* 2023). Soybean yield is extremely sensitive to abiotic stresses such as cold, heat, drought, salt, and heavy metals. A thorough understanding of soybean responses to abiotic stresses at the genetic level is a prerequisite for breeding stress-tolerant cultivars. Compared to the roles of miRNAs in the responses to biotic stresses in soybean, the functions of miRNAs in the plant’s response to abiotic stress are much better understood. Here, we summarized the known regulatory mechanisms by miRNAs on soybean growth under nutrient starvation, low and high temperatures, drought, and salt stress.

#### Nutrient starvation

Plant miRNAs are considered the principal regulators of responses to low nutrient availability and are vital for maintaining nutrient homeostasis through regulating the expressions of nutrient transporters (Paul *et al.* 2015). Phosphorus (P) deficiency is the major nutrient constraint on crop production (Jiang *et al.* 2021). Several research groups have carried out the genome-wide identification of soybean miRNAs in response to P deficiency (Sha *et al.* 2012, Xu *et al.* 2013, Zhang *et al.* 2021). Their results showed that miR399 was highly upregulated in soybean roots under P deficiency and was a key regulator of phosphate (Pi) uptake (Fan *et al.* 2021, Sha *et al.* 2012, Xu *et al.* 2013, Zhang *et al.* 2021). *PHO2* (*PHOSPHATE 2*) encodes an E2 ubiquitin-conjugating enzyme and is a known target of miR399 (Aung *et al.* 2006, Bari *et al.* 2006, Du *et al.* 2018, Fan *et al.* 2021) as well as a negative regulator of *PHT1* (*PHOSPHATE TRANSPORTER 1*), a transporter responsible for Pi uptake (Bari *et al.* 2006, Du *et al.* 2018). The P starvation-induced miR399 repressed the expression of *PHO2* and de-repressed *PHT1* expression, resulting in an increased Pi uptake from soil (Bari *et al.* 2006).

Different members in the miR156 family showed divergent expression patterns during soybean nodulation under low nitrogen conditions (Yun *et al.* 2022). The expression of miR156b was sharply reduced at 1 DAI and then more gradually downregulated over the duration of the experiment from 1–5 DAI, displaying an opposite expression pattern to miR172c, a positive regulator of nodulation (Yun *et al.* 2022). *SQUAMOSA* *promoter-binding protein-like 9d* (*SPL9d*) is verified as a target gene of miR156b (Yun *et al.* 2022). The miR156b-*SPL9d* module is an upstream master regulator of soybean nodulation (Yun *et al.* 2022). Therefore, nitrogen deficiency could increase soybean symbiosis with rhizobia (Gibson and Pagan 1977). *SPL9d* directly binds to the promoters of *miR172c*, *nodule inception a* (*NINa*), and *ENOD40-1* to activate their transcription (Yun *et al.* 2022). Since NINa, ENOD40-1, and miR172c are all positive regulators of nodulation, the binding of *SPL9d* to their promoters ensures the soybean plant can acquire additional nitrogen from symbiotic nitrogen fixation in nodules to compensate for the diminished uptake from nitrogen-deficient soil (Charon *et al.* 1997, Fan *et al.* 2023, Wang *et al.* 2019, Yun *et al.* 2022). On the other hand, miR156b negatively regulates nodule formation by targeting the positive regulator of nodulation, *SPL9d* (Yun *et al.* 2022). The dynamic nature of miR156 induction by low nitrogen in the soil and its roles as both a promoter of nodulation and a negative regulator of nodule formation, depending on its expression level and the degree of nodulation.

Sulfur is a critical macronutrient for soybean production. Sulfur deficiency limits soybean growth and inhibits the biosynthesis of rhizobium nitrogenase and soybean proteins (Hu *et al.* 2023). Under sulfur starvation treatment, miR395 was highly induced in Arabidopsis, and was the key miRNA responsible for increased sulfur uptake and translocation by targeting the genes of the plastid ATP sulfurylase, *APS1*, and the sulfate transporters, *SULTR2;1* and *SULTR2;2* (Liang *et al.* 2010). A high expression level of miR395 in Arabidopsis led to the over-accumulation of sulfur in shoots, while a reduced expression of miR395 resulted in the decline in sulfate accumulation (Ai *et al.* 2016). MiR395 is identified in soybeans and targets soybean *SULTR2*, Glyma.08G138600 and Glyma.18G168900 (Goettel *et al.* 2014).

#### Heat/cold stress

Chilling and heat stresses are major abiotic factors inhibiting soybean yield in temperate climate (Kumar 2014, Xu *et al.* 2016, Zhang *et al.* 2014). Cold-responsive miRNAs and their target genes, along with small-RNA transcriptomes, were studied in soybean roots, leaves, shoots and nodules (Kuczyński *et al.* 2022, Xu *et al.* 2016, Zhang *et al.* 2014). Upon chilling stress, miR1508a was induced and was found to control the expressions of the *pentatricopeptide repeat* gene (*PPR*, *Glyma.16G162100*) and *xyloglucan endo-trans-glucosylase/hydrolase* (*XTH*, *Glyma.17G065400*) (Sun *et al.* 2020). The *miR1508a*-transgenic soybean showed improved cold tolerance during the germination and early seedling stages and had 58% higher soluble sugar content than the wild type when subjected to cold treatment (Sun *et al.* 2020). PPR proteins are responsible for the maturation of organelle RNA in eukaryotic organisms and targeting the orthologous NADH dehydrogenase complex in the mitochondrion and the NADH dehydrogenase-like complex in the chloroplast (Rovira and Smith 2019). NADH dehydrogenase is well known to be involved in the light reaction of photosynthesis, the Calvin cycle, photorespiration and the photosynthetic adjustments in response to environmental stresses (Fromm *et al.* 2016, Ruhlman *et al.* 2015). Therefore, the cold tolerance conferred by the overexpression of miR1508a in soybeans may be due to its indirect participation in photosynthesis and respiration through controlling the expression of *PPR* (Sun *et al.* 2020). In Arabidopsis, *ppr* deletion mutants exhibited severe growth delay (Waltz *et al.* 2019). XTH promotes cell wall elongation (Miedes *et al.* 2013). The stunted phenotype of *miR1508a*-transgenic soybean plants may be due to the downregulation of *XTH* and *PPR* by miR1508a (Sun *et al.* 2020).

Many heat-responsive miRNAs, such as miR156, have been found in legumes (Matthews *et al.* 2019). In alfalfa (*Medicago sativa*), the expression level of miR156 was significantly induced after 30 and 120 min of heat stress. The overexpression of miR156 in alfalfa enhanced its heat tolerance to up to 40°C, with higher water potential, and increased anthocyanin and chlorophyll abundance (Matthews *et al.* 2019). However, the whole miR156 family was repressed in soybean after heat treatment for 7 d (Ding *et al.* 2021). Moreover, the overexpression of soybean miR156b in Arabidopsis led to heat stress damage phenomena, such as yellowing and wilting of some flowers. The wild-type Arabidopsis line showed to be normal under moderate heat stress (Ding *et al.* 2021).

#### Salinity and drought stress

Salinity greatly restricts the soybean yield in arid and semi-arid lands (Guan *et al.* 2024). MiRNAs, including miR156b, miR160a, miR390, miR394a, miR4413a, miR4416c and miR5225, showed divergent expression trends in soybean from 0 to 72 h of salt treatment (Li *et al.* 2022b). Some miRNAs, such as miR156r, had lower expression levels from 4 to 24 h of salinity stress than in the 0-h control (Li *et al.* 2022b). On the other hand, soybean miR172a was induced by salt and drought treatments (Pan *et al.* 2016). Transgenic soybean hairy roots overexpressing miR172a had better growth and higher survival rates than control plants under salt stress (Pan *et al.* 2016). Another member of the miR172 family, miR172c, is a negative regulator of salt tolerance and increases the root sensitivity to salt stress (Sahito *et al.* 2017, Wang *et al.* 2019). Ectopic expression of miR4359b improved root development, plant survival rate and chlorophyll content of soybean under salt stress, while the miR4359b-knockdown mutant had enhanced salt sensitivity (Yu *et al.* 2023). Three miRNAs, miR169c, miR393a, and miR398c, negatively regulate drought tolerance in soybean (Xing *et al.* 2023, Yu *et al.* 2019, Zhou *et al.* 2020). Overexpressing the soybean miR169c and miR398c in *Arabidopsis* increased plant sensitivity to drought stress, leading to a low survival rate and accelerated leaf water loss (Yu *et al.* 2019, Zhou *et al.* 2020). Loss-of-function analyses of miR393a in soybean demonstrated reduced drought tolerance in terms of root vigor, net photosynthetic rate, and relative water content (Xing *et al.* 2023).

#### Responses to multiple stresses

In recent years, more and more miRNAs have been found to respond to multiple stresses in soybean (Fan *et al.* 2023, Ning *et al.* 2019). Twenty-one miRNAs, including miR169l-3p, miR5036, miR862a, and miR398a/b, were found to be responsive to the combined Pi starvation and salinity stress (Ning *et al.* 2019). Six miR159 genes, namely *miR159a-f*, responded differently from one another when under P deficiency, high salinity, and abscisic acid treatments (Li *et al.* 2023). The overexpression of miR159e decreased the total P content in soybean leaves under low P conditions and enhanced soybean resistance to *H. glycines* (Lei *et al.* 2021, Li *et al.* 2023). Besides cold stress, miR1508a was also inhibited by drought stress, and overexpressing it resulted in a dwarf phenotype and reduced drought resistance in soybean (Sun *et al.* 2020). The multifunctional characteristics of miRNAs in regulating soybean phenotypes and tolerance to multiple stresses make miRNAs an effective crop breeding target.

## Histone modifications associated with soybean stress tolerance

On top of signaling pathways and transcription factor networks, histone modifications add another layer of regulation to the transcription of stress-responsive genes (Kang *et al.* 2022, Liu *et al.* 2022b). In this section, the latest discoveries on the roles histone modifications play in soybean stress responses are discussed (Tables 2, 3).

### Biotic stress

The studies on the involvement of histone modifications in soybean-pathogen interactions have mainly focused on *Phytophthora sojae*, which is an oomycete pathogen that infects soybean root (Tyler 2007). During infection, *P. sojae* secretes host cell-penetrating effector proteins to disrupt plant immunity. Histone modifications are important components in plant immunity through their regulations of defense-related genes. Histone acetylation facilitates gene transcription, and their levels in defense-related soybean genes can be manipulated by two RxLR effectors, PsAvh23 and PsAvh52, during *P. sojae* infection (Kong *et al.* 2017, Li *et al.* 2018). PsAvh23 competitively binds to GmADA2 (*Alteration/Deficiency in Activation 2*) and disrupts the formation of the ADA2-GCN5 (General Control Non-depressive 5) subcomplex, leading to a reduction in the levels of GmGCN5-mediated H3K9ac at the defense genes and thus their downregulation (Kong *et al.* 2017). At the same time, PsAvh52 promotes the relocation of the acetyltransferase, GmTAP1, into the nucleus, allowing GmTAP1 to acetylate the histone H2A and H3 of the susceptibility genes and activate their transcription, therefore enhancing soybean susceptibility to *P. sojae* (Li *et al.* 2018). Furthermore, silencing *GmADA2* or *GmGCN5* increased soybean susceptibility to *P. sojae* whereas *GmTAP*-silenced soybean was more resistant to *P. sojae* (Kong *et al.* 2017, Li *et al.* 2018).

Histone modifications in the pathogen genomes are also important in determining the virulence of the pathogens. A recent study showed that the expression of *Avr1b* was affected by the level of histone methylation (Wang *et al.* 2020). When the histone methyltransferase *PsSu(z)12* was knocked out in *P. sojae*, the level of H3K27me3 at *Avh1b* decreased and the expression of *Avh1b* increased, so the *PsSu(Z)12* mutants could no longer infect the *Rps1b* soybean cultivars (Wang *et al.* 2020). By knocking out another histone methyltransferase, *PsKMT3*, the H3K36me3 level was reduced and a majority of the RxLR effectors were downregulated, thus reducing the pathogenicity of *P. sojae* (Chen *et al.* 2023). The *P. sojae* mutants with silenced histone acetyltransferase, *PsGCN5*, also had reduced virulence towards soybean, probably due to the impaired ability to suppress reactive oxygen species (ROS) production in soybean, enabling the plant to mount an early immune response (Zhao *et al.* 2015).

### Abiotic stress

#### High/low temperature stress

The changes in histone modifications in response to abiotic stress have been well documented in soybean (Yung *et al.* 2023). Genome-wide histone methylation and acetylation are reported to be responsive to changes in temperature (Stępiński 2012, Yang *et al.* 2018). The increase in H3K4me2 and decrease in H3K4me3 and H3ac were observed both in soybean roots under chilling condition at 10°C and in soybean leaves under cold stress at 4°C (Stępiński 2012, Yang *et al.* 2018). When soybean leaves were subjected to heat stress at 42°C, the level of H3ac increased while both the levels of H3K4me2 and H3K4me3 remained unchanged (Yang *et al.* 2018).

#### Drought and salinity stress

The activation of transcription factors is essential to trigger soybean stress tolerance mechanisms. It is therefore important to understand the regulation of stress-responsive transcription factors. The histone modification status was examined in selected salt-inducible transcription factors, and the hypo-H3K4me2, hyper-H3K4me3, and hyper-H3K9ac at the loci of *Glyma.11g02400*, *Glyma.20g30840*, and *Glyma.08g41450* indicated that histone methylation and acetylation are involved in the induction of transcription factors under salt stress (Song *et al.* 2012). Furthermore, a higher level of global H3K27me3, most likely due to the *de novo* H3K27me3 marks that are only found in the salt-treated samples, was observed in soybean roots under salt stress (Sun *et al.* 2019), while half of the genes with a lower H3K27me3 level under high-salt conditions were also upregulated, including the salt-responsive genes *Glyma.07g110300*, *Glyma.04g131800*, and *Glyma.04g187000*, suggesting that the H3K27me3 mark plays a role in transcription regulation in soybean in reaction to high salinity (Sun *et al.* 2019).

It has been proposed that epigenetic changes are the underlying mechanisms of stress memory in plants (Lämke and Bäurle 2017). In soybean, the profiles of H3K4me2, H3K4me3, and H3K9ac underwent extensive changes in both leaves and roots during salt stress priming, a process of pre-treating soybean seedlings with low salinity before subjecting them to a subsequent high-salt stress (Yung *et al.* 2022). The changes in histone modifications were highly correlated with the altered transcriptional responses in the primed soybean, resulting in enhanced osmotic stress signaling and ion homeostasis and the repression of defense-related genes, culminating in higher salt tolerance in the primed soybean plants than the unprimed controls (Yung *et al.* 2022).

#### Histone-modifying enzymes

The roles of histone-modifying enzymes in soybean stress responses have also been investigated (Table 3). The expression of histone deacetylases (HDACs) in soybean subjected to different abiotic stresses were examined (Yang *et al.* 2018). Under temperature or osmotic stress, the expressions of most *GmHDACs* were downregulated, while *GmSRT2* (*NAD-DEPENDENT PROTEIN DEACETYLASE SIRTUIN-4*) was significantly induced by cold, heat, and NaCl treatments (Yang *et al.* 2018). A group of histone demethylases, *GmLDLs* (*LYSINE-SPECIFIC HISTONE DEMETHYLASE-LIKEs*), also demonstrated stress-responsive expression patterns under different environmental conditions (Liu *et al.* 2022a). The time-course expression profiles of *E2 ubiquitin-conjugating enzymes* (*UBC*s) in soybean seedlings in response to drought showed that at least six *GmUBC*s were upregulated (Chen *et al.* 2020). The most highly induced among them was *GmUBC9*, suggesting that it possesses an important function in soybean drought stress response, which was confirmed by the enhanced drought tolerance in transgenic soybean overexpressing *GmUBC9* (Chen *et al.* 2020).

It was recently revealed that, upon salt stress, the histone acetyltransferase, GsMYST1, is phosphorylated by GsSnRK1 (SUCROSE NON-FERMENTING 1-related PROTEIN KINASE 1), and the GsMYST1-GsSnRK1 complex is then recruited by a transcription factor GsNAC83 to the promoter of *COR15B* (*COLD-REGULATED 15B*), resulting in an increase in the H4ac level at the promoter and the subsequent transcript level of *COR15B* (Feng *et al.* 2022a). It was also observed that only those soybean hairy roots transformed with both *GsSnRK1* and *GsMYST1* demonstrated enhanced salt tolerance, indicating that both the phosphorylating function of GsSnRK1 and the acetyltransferase function of GsMYST1 are essential for soybean salt tolerance (Feng *et al.* 2022a).

GmZF351, a CCCH-type zinc finger protein, is activated upon salt and osmotic stress to trigger stomatal closure, by regulating the expressions of a CBL-interacting serine/threonine protein kinase *GmCIPK9* (*Glyma.17g066300*) and a serine/threonine protein kinase *GmSnRK* (*Glyma.02G135500*). The activation of *GmZF351* is mediated through a reduced H3K27me3 level at its promoter and two JMJ30 demethylases were identified to be responsible for this demethylation process (Wei *et al.* 2023). Transgenic hairy roots overexpressing *GmJMJ30-1* or *GmJMJ30-2* had better stress tolerance and higher water content under mannitol or NaCl treatments (Wei *et al.* 2023).

The accumulation of GmNFYA (NUCLEAR FACTOR Y subunit) under salt stress prevents the binding of a histone deacetylase GmHDA13 to a WD40-repeat family protein GmFVE, thus maintaining the H3K9ac level at the promoters of stress-responsive genes such as *GmLOS2* (*LOW EXPRESSION OF OSMOTICALLY RESPONSIVE GENES 2*), *GmXTH-23* (*xyloglucan endotransglucosylase/hydrolase 23*), and *GmOMT1* (*O-methyltransferase 1*) (Lu *et al.* 2021). The role of GmHDA13 as a negative regulator of salt tolerance in soybean was confirmed by the increased salt tolerance in *GmHDA13*-RNAi transgenic lines and heightened salt sensitivity in *GmHDA13*-overexpressing transgenic hairy roots (Lu *et al.* 2021).

#### Readers of histone marks

The recognition of histone marks by specific readers further increases the complexity of the gene regulatory mechanism involving histone modifications (Table 3). A salt-inducible plant homeodomain finger protein, GmPHD5, preferentially recognizes H3K4me2 and can interact with the acetyltransferase, GmGNAT1, the transcription elongation factor, GmElongin A, and the chromatin-remodeling protein, GmISWI (Wu *et al.* 2011). The binding of GmPHD5 to the promoters and gene bodies of *GmRD22* (*RESPONSIVE TO DESICCATION 22*) and *GmGST* (*glutathione S-transferase*) illustrates the putative role of GmPHD5 in regulating the expressions of stress-related genes by recruiting proteins involved in transcription (Wu *et al.* 2011). Meanwhile, GmPHD6 recognizes the H3K4me0/1/2 marks and binds to the promoters of *CYP71A22*, *ASR* (*ABA-stress ripening*), and *CYP82C4* to activate their transcriptions by recruiting the transcription activator, GmLHP1 (like-heterochromatin protein 1). The three target genes together facilitate stomatal closure under salt stress and confer tolerance to salinity (Wei *et al.* 2017). The enhanced salt tolerance in the *GmPHD6*-overexpressor and enhanced salt sensitivity in the *GmPHD6*-RNAi transgenic hairy roots further support the role of *GmPHD6* in soybean salt tolerance (Wei *et al.* 2017).

## Future perspectives

Global soybean yield has been largely affected by biotic and abiotic stresses. As previously reported, open-air experiments predicted that climate changes would result in a 20% decline in soybean production (Morgan *et al.* 2003). The United States is one of the major soybean producers in the world, with a yield of 4.2 billion bushels in 2023 (Sisson 2024); however, the yield was reduced by around 1.6% due to invertebrate pests in that year (Sisson 2024). Therefore, improving soybean stress resistance becomes a major breeding goal. Since miRNAs and histone modifications are highly related to soybean stress tolerance, the application of miRNAs and the manipulation of histone modifications would be effective ways to increase soybean yield in the face of environmental stresses.

### Applications of miRNAs in soybean breeding to enhance stress tolerance

Extensive research has revealed that miRNAs are critical regulators of soybean stress tolerance and provide valuable genetic resources for molecular breeding. Short tandem target mimic (STTM) and clustered regularly interspaced short palindromic repeats (CRISPR) are two widely used approaches in molecular breeding using miRNAs. The STTM structure includes two miRNA-binding sites and can efficiently reduce the abundance of mature miRNAs (Tang *et al.* 2012). The STTM construct can be applied in a constitutive, inducible or tissue-specific module driven by the corresponding promoter to precisely edit the crop trait of interest (Chen *et al.* 2021). The pivotal role of miR166 in determining plant height was revealed in soybean using an STTM-mediated *miR166*-knockdown line (Zhao *et al.* 2022). To avoid the public debates about genetically modified organisms (GMOs), CRISPR/Cas9 (CRISPR activation/endonuclease deficient Cas9) is widely used in crops as a transgene-free way of editing crop genomes. To improve the soybean yield, six *miR396* genes were knocked out by CRISPR in the elite soybean cultivar ‘Zhonghuang 302’ (Xie *et al.* 2024). Two triple mutants, *miR396adf* and *miR396cdf*, have significantly increased the seed size and yield in the field (Xie *et al.* 2024). This research confirmed the valuable potential of using miRNAs in soybean breeding programs and provided a practical protocol.

Exogenous miRNAs and small interfering RNAs (siRNAs) can be taken up by plants to activate their RNA interference (RNAi) machinery of post-transcriptional gene silencing (Betti *et al.* 2021). Therefore, plant miRNAs, which function as positive regulators for environmental stress responses, can be artificially synthesized and directly applied to soybeans to specifically enhance soybean stress tolerance and nutrient uptake. In one study, RNA was extracted from *miR399/miR156*-overexpressing Arabidopsis plants into a liquid medium in which wild-type Arabidopsis seedlings were cultivated. After a 24-h incubation, *PHO2* (the miR399 target) and *SPL9* (the miR156 target) were downregulated compared to the untreated wild-type control, showing that this treatment could directly enhance plant phosphate uptake and stress tolerance (Betti *et al.* 2021). This evidence highlights the potential usage of exogenous miRNA in altering plant phenotypes.

The artificial miRNA (amiRNA) technology was an effective tool for silencing specific genes in plants (Schwab *et al.* 2006), and the exogenous application of amiRNA in a soybean breeding program will allow the breeder to achieve the phenotypic goal for the crop while sidestepping the controversies surrounding GMOs. In one application, the amiRNA technique was employed to reduce insect attacks in Arabidopsis (Gualtieri *et al.* 2020). The amiRNAs produced by engineered plants were transferred into insect pests during feeding to specifically inhibit their target mRNAs in the insects and therefore reduce the infestation and damage to the crop (Gualtieri *et al.* 2020). In another example, an engineered rice expressing the amiRNA of the endogenous csu-novel-miR260 of striped stem borer (SSB; *Chilo suppressalis*) gained resistance to SSB (Zheng *et al.* 2021). Hence, plant breeding using miRNAs could be an effective approach for sustainable crop production.

### Enhancing soybean stress tolerance through manipulating histone modifications

Since certain histone modifications have been found to be associated with abiotic and biotic stress responses (as discussed in a previous section), manipulating them could have the potential to enhance soybean stress tolerance. One way of manipulation is through the use of chemical inhibitors of histone-modifying enzymes. These inhibitors have been used in research to demonstrate the functions and roles of histone-modifying enzymes in regulating stress responses in plants, so they could be utilized on these enzymes in field applications to increase soybean stress tolerance. It is worthwhile noting that most of these chemical inhibitors are inhibitors of histone deacetylases (HDACs), which have been shown to be key epigenetic factors in repressing gene expressions in plant growth, development, and responses to abiotic and biotic stresses (Yang *et al.* 2018).

The addition of Ky-2, a Class-I histone deacetylase inhibitor, to *A. thaliana* led to an elevation in the global histone acetylation level, and an increase in the expressions of salt-responsive genes, including *AtSOS1* (*salt overly sensitive 1*) and *AtP5CS1* (*Delta-1-pyrroline-5-carboxylate synthase*) (Sako *et al.* 2016). The upregulation of *AtSOS1* and *AtSOS3* under Ky-2 treatment facilitated the exclusion of Na^+^ ions and improved salt tolerance in Arabidopsis (Sako *et al.* 2016).

Suberoylanilide hydroxamic acid (SAHA) treatment led to the hyperacetylation of histones H3 and H4 in the roots of cassava (*Manihot esculenta*) and contributed to the upregulation of *MeSOS1*, enhancing salt tolerance in the crop (Patanun *et al.* 2017). Moreover, the application of Trichostatin A (TSA) in soybean induced the H3K9ac levels of *GmCLC1*, *ARF16-related*, *PP2C68-related*, and *SCR-related* genes, leading to an increase in the transcriptional response of the priming-induced genes and higher photosynthetic performance under salt stress (Yung *et al.* 2022).

The application of sodium butyrate (NaBT) to rice (*Oryza sativa*) caused a drastic increase in the global H3K9ac level, de-repressing the defense-related genes, including *Osbsr-d1*, *OsPR10B*, *OsNAC4* and *OsKS4*, and increased the immunity of rice against *Magnaporthe oryzae* (rice blast disease) (Xu *et al.* 2022a).

In tomato, the application of sodium valproate (SV), an HDAC inhibitor, enhanced the acetylation of histone H3 in the fungal pathogen, *Botrytis cinerea*, including H3K9ac, H3K14ac, and H3K56ac, upregulating the histone H3 acetylation-mediated gene transcriptions (Xu *et al.* 2022b). Nonetheless, SV lowered the expressions of virulence genes and altered the expressions of ROS response genes of *B. cinerea*, lowering the infection efficiency of *B. cinerea* against the tomato plant as a result (Xu *et al.* 2022a). SV also triggered the immune response of tomato against *B. cinerea*, enhancing the resistance of tomato against the pathogen infection (Xu *et al.* 2022a).

Aside from HDAC inhibitors, the plant regulator coronatine, an analog of jasmonic acid (JA), was also demonstrated to increase the cold tolerance of tomato (Liu *et al.* 2022c). The application of coronatine elevated the global H3K4me3 level and upregulated the master transcription factor genes, chilling-related genes, and jasmonic acid-related genes (Liu *et al.* 2022c). The extensive transcriptional reprogramming allowed tomato to acquire a higher chilling tolerance (Liu *et al.* 2022c).

Despite the evidence of tolerance enhancement in various plants through chemicals, the molecular mechanisms of how these chemicals and epigenetic modifications work together to increase soybean tolerance under different abiotic and biotic stresses have not been fully understood. More in-depth studies are needed to understand the epigenetic mechanisms of stress responses in soybean and to find the most appropriate chemicals that have the fewest undesirable side effects for enhancing soybean tolerance against stresses.

Besides employing enzyme-inhibiting chemicals to produce stress-tolerant soybean, breeders can also carry out targeted epigenome editing through CRISPR/Cas9. In *A. thaliana*, the application of CRISPRa/dCas9 fused with a histone acetyltransferase enhanced the acetylation of Lysine 27 of Histone 3 (H3K27ac), thereby upregulating *AREB1* (*ABSCISIC ACID-RESPONSIVE ELEMENT BINDING PROTEIN 1*), leading to a stronger drought stress response (Paixão *et al.* 2019). The technological advancement in targeted epigenome editing provides possibilities for enhancing soybean tolerance towards abiotic and biotic stresses through histone modifications. However, most studies on targeted epigenome editing used *A. thaliana* as the model organism. Therefore, more studies have to be carried out to better understand the histone modifications associated with stress responses in soybeans. Also, epigenetic editing tools have to be developed and tailored for use in soybean before they can be utilized in breeding programs.

## Author Contribution Statement

H-ML designed the conceptual framework of this paper and coordinated the writing. KF and LW co-wrote the sections on the microRNA effects and applications in soybean stress tolerance. L-YC, S-WC, W-SY, and HZ co-wrote the sections on histone modification effects and applications in soybean stress tolerance. KF, W-SY, and H-ML revised and polished the article.

## Figures and Tables

**Table 1.** MiRNAs that are differentially expressed in soybean under different stresses

MiRNA family	Target genes	Biotic stress	Abiotic stress
miR160	*ARF*	*Pseudomonas syringae* (Fahlgren *et al.* 2007)	Salt stress (Li *et al.* 2022b)
miR164	*NAC*	*Alternaria brassicicola* and *Pseudomonas syringae* (Lee *et al.* 2017)	Salt and drought stress (Hernandez *et al.* 2020)
miR166	*HD-ZIPIII*	*Phytophthora sojae* (Wong *et al.* 2014)	Salt stress (Li *et al.* 2017)
miR167	*ARF*	*Hibiscus cannabinus* L. (Gao *et al.* 2013), and Soybean mosaic virus (Yin *et al.* 2013)	Alkaline stress (Lv *et al.* 2012)
miR168	*AGO1*	Soybean mosaic virus (Chen *et al.* 2015)	Heat stress (Zhou *et al.* 2016)
miR390	*TAS3*	Soybean mosaic virus (Li *et al.* 2022a)	Salt stress (Li *et al.* 2022b)
miR393	*TIR1*	*Phytophthora sojae* (Wong *et al.* 2014)	Drought stress (Xing *et al.* 2023)
miR396	*GRF*	*Heterodera glycines* (Noon *et al.* 2019)	Drought stress (Xie *et al.* 2024)
miR398	*CSD*	*Heterodera glycines* (Tian *et al.* 2017)	Drought stress (Zhou *et al.* 2020)
miR482	*NBS-LRR*	*Verticillium dahilae* (Zhu *et al.* 2013)	Cold stress (Kuczyński *et al.* 2022)
miR156	*SPL*	Soybean mosaic virus (Bao *et al.* 2018)	Heat stress (Ding *et al.* 2023)
miR159	*GAMYB*	*Heterodera glycines* (Lei *et al.* 2021)	Phosphate starvation, Salt and ABA treatment (Li *et al.* 2023)
miR172	*AP2*	Rhizobium (Sahito *et al.* 2017, Yan *et al.* 2013)	Drought and Salt (Pan *et al.* 2016)
miR399	*PHO2*	*Heterodera glycines* (Tian *et al.* 2017)	Phosphate starvation (Fan *et al.* 2021)
miR4407	*IPT3*	Rhizobium (Fan *et al.* 2023)	Salt stress (Fan *et al.* 2023)
miR319	*TCP*	*Heterodera glycines* (Li *et al.* 2012)	Cold stress (Kuczyński *et al.* 2020)
miR408	*LACCASES*	*Heterodera glycines* (Feng *et al.* 2022b)	Drought stress (Hajyzadeh *et al.* 2015)
miR1507	*NB-LRR*	*Phytophthora sojae* (Wong *et al.* 2014)	Cold stress (Kuczyński *et al.* 2022)
miR1508	*PPR*, *XTH*		Cold and drought stress (Sun *et al.* 2020)
miR1510	*NB-LRR*	*Phytophthora sojae* (Cui *et al.* 2017)	
miR2109	*NB-LRR*	*Phytophthora sojae* (Wong *et al.* 2014)	
miR1520	*Glyma.19G182000*	*Heterodera glycines* (Khoei *et al.* 2021)	
miR5770	*Glyma.17G019300* *Glyma.03G005400*	*Heterodera glycines* (Khoei *et al.* 2021)	
miR5032	*Glyma.05G180700* *Glyma.08G138300*	*Heterodera glycines* (Khoei *et al.* 2021)	
miR5043	*Glyma.13G270900*	*Heterodera glycines* (Khoei *et al.* 2021)	

**Table 2.** Histone modifications that are responsive to different stresses in soybean

Histone modifications	Histone modification regulators	Biotic stress	Abiotic stress
H3K9ac	*GmGCN5*	*Phytophthora sojae* (Kong *et al.* 2017)	
H2Aac H3ac	*GmTAP1*	*Phytophthora sojae* (Li *et al.* 2017)	
H3K4me2 H3K4me3 H3ac			Chilling (10°C) and cold stress (4°C) (Stępiński 2012, Yang *et al.* 2018)
H3ac			Heat stress (Yang *et al.* 2018)
H3K4me2 H3K4me3 H3K9ac H3K27me3			Salt stress (Song *et al.* 2012, Sun *et al.* 2019, Yung *et al.* 2022)

**Table 3.** Histone-modifying enzymes and readers of histone marks that are responsive to different abiotic stresses in soybean

Histone-modifying enzymes	Readers of histone marks	Types of abiotic stress
GmHDACs, GmSRT2		Cold, heat, flooding, drought, and salt stress (Yang *et al.* 2018)
GmSRT2		Cold, heat and salt stress (Yang *et al.* 2018)
GmLDLs		Shade, cold, heat, flooding, drought, salt and low Pi stress (Liu *et al.* 2022a)
GsMYST1		Salt stress (Feng *et al.* 2022a)
GmHDA13		Salt stress (Lu *et al.* 2021)
	GmPHD5	Salt stress (Wu *et al.* 2011)
	GmPHD6	Salt stress (Wei *et al.* 2017)
